# Effect of perioperative magnesium sulfate and labetalol infusion on peripheral perfusion and postoperative pain in nasal surgery: a randomized controlled trial

**DOI:** 10.1186/s13037-022-00336-7

**Published:** 2022-08-19

**Authors:** Alshaimaa Abdel Fattah Kamel, Marwa Mohamed Medhat, Dina Abdelhameed Elsadek Salem, Sara Mohamed Abdel Naby

**Affiliations:** Anesthesia, Intensive Care and Pain Management Department, Faculty of Human Medicine, Zagazig University, Alsharkia, Egypt

**Keywords:** Peripheral perfusion index, Magnesium sulfate, Labetalol, Postoperative Pain, Induced hypotension, Nasal surgery

## Abstract

**Background:**

Maintenance of adequate peripheral perfusion during controlled hypotension is necessary for patient safety and improved surgical outcomes during controlled hypotension in nasal surgery. The hypothesis of this study was to investigate the effect of perioperative magnesium sulfate and labetalol infusion on peripheral perfusion and postoperative pain in patients undergoing nasal surgery.

**Methods:**

A total of 50 patients were randomly assigned into two equal groups in this double-blind clinical study: the magnesium sulfate group; received 40 mg/kg loading dose of intravenous (IV) magnesium sulfate followed by 10–15 mg/kg/h continuous IV infusion and the labetalol group; received 0.25 mg/kg loading dose of IV labetalol followed by 0.5–1 mg/kg/h continuous IV infusion to achieve a mean arterial blood pressure (MABP) of = 55–65 mmHg. The primary outcome was to compare the effect of perioperative magnesium sulfate and labetalol infusion on peripheral perfusion during nasal surgery. The secondary outcomes were the assessment of serum lactate, postoperative pain, time to the first call for pethidine (rescue analgesic) and total pethidine consumption.

**Results:**

PPI was comparable between the groups at baseline, intubation, and 5 min. In contrast, magnesium sulfate group had a significantly higher PPI than the labetalol group. The magnesium sulfate group had a significantly higher MABP and heart rate compared to labetalol group. The time to reach the target MABP was significantly prolonged in magnesium sulfate than the labetalol group [21.6 ± 1.7 vs 6.9 ± 1.5] min. VAS scores were significantly lower for 2 hs postoperatively in the magnesium sulfate group than the labetalol group. The time to first call of pethidine was significantly prolonged in the magnesium sulfate group compared to the labetalol group [113.1 ± 5.2 vs 28.2 ± 1.5] min.

**Conclusions:**

Magnesium sulfate maintains wider PPI and offers better postoperative pain relief compared to labetalol during induced hypotension in nasal surgery.

**Trial registration:**

Institutional review board approval (ref: 6601/20–12-2020).

Clinicaltrial.gov (ref: NCT04688203, date of registration: 29 -12–2020).

**Supplementary Information:**

The online version contains supplementary material available at 10.1186/s13037-022-00336-7.

## Background

Impaired peripheral perfusion and consequent vital organ ischemia are considered major threats to hypotension [1]. Achieving a target mean arterial blood pressure of 55–65 mmHg during surgery is adequate to produce a bloodless surgical field, but may not be sufficient for adequate oxygen supply to various organs [1, 2].

As monitoring of perfusion to vital organs is complicated and invasive, monitoring of peripheral perfusion of non-vital organs such as the skin is sufficient [3]. Moreover, measurement of the peripheral perfusion index (PPI) by pulse oximetry is a simple, beneficial, and noninvasive method [4].

PPI is the ratio of infrared pulsatile signals to the non-pulsatile signals and is expressed as a percentage. The normal values range from 0.02% to 20%. Maintaining PPI at optimal levels during surgery leads to quicker and smoother recovery of the patients, with fewer postoperative complications and better surgical outcomes [5, 6]. Additionally, effective treatment of pain after nasal surgery enhances recovery and improves surgical outcomes [7].

Magnesium sulfate is a reliable drug that induces hypotension, and its analgesic effect has been studied in many literatures over the last few decades by antagonizing the N-methyl-D aspartate receptor [8–10].

Labetalol is another hypotensive agent that acts through competitive inhibition of α and B- adrenergic receptors [currently, and beta- adrenergic receptors are a promising target for pain management as they are distributed in the nervous system (B2 > B1 > B3)]. B2 adrenergic receptors are found in peripheral nociceptors and in the spinal cord sensitizing nociceptors and enhancing pain signals by the existence of proinflammatory cytokines [11, 12].

Thus, this study aimed to compare the effects of magnesium sulfate and labetalol infusion during induced hypotension on peripheral perfusion and postoperative pain in nasal surgeries. The primary outcome was to compare the effect of perioperative magnesium sulfate and labetalol infusion on peripheral perfusion during nasal surgeries. The secondary outcomes were the assessment of serum lactate**,** postoperative pain, time to the first call for pethidine (rescue analgesic**)** and total pethidine consumption.

## Methods

### Study design and population

This prospective randomized double-blind clinical study was approved by the Institutional Review Board of our university (ref: IRB#6601/20–12-2020) and was registered at clinicaltrial.gov (ref: NCT04688203, date of registration: 29 -12–2020) https://clinicaltrials.gov/ct2/show/NCT04688203 prior to patient enrollment. Written informed consent was obtained from all patients participating in this trial. The first patient enrolled at 1–2-2021. The current study adheres to CONSORT guidelines and includes a completed CONSORT checklist.

This was a double-blind study, as both the patient and the outcome assessors (physician anesthesiologist collecting the data) were blinded to the study drugs. It was conducted from 1 February 2021 to 30 September 2021 on 50 patients of either sex, belonging to the American Society of Anesthesiologist (ASA) I and II physical status, aged between 21 and 45 years with a body mass index (BMI) ranging from 20 to 30 kg/m^2^ and undergoing elective nasal surgeries such as septoplasty, functional endoscopic sinus surgery and septoturbinoplasty. Duration of surgery was ≤ 2 h. Patients on beta-blocker or anticoagulants or receiving pain killer, who are diabetic, and asthmatic, with advanced (renal, hepatic, respiratory, cardiovascular) diseases, with an altered mental state, and known allergy to drugs were excluded from the study.

A routine clinical evaluation performed preoperatively for all patients. All patients were kept nil orally for 8 h for a heavy meal and 2 h for clear fluids preoperatively and were learned the visual analogue scale (VAS) score [13]. It is a 10 cm line with 10 as the worst and 0 with no pain. An intravenous (IV) line was inserted, midazolam 0.03 mg/kg was administered and warm fluids were started at a rate of 5 mL/kg/h. The patients were transferred to the operating room and standard monitors were connected to the patients: such as electrocardiogram, non-invasive blood pressure and pulse oximeter. Warm blankets were used and the operating room temperature was adjusted to 25 ˚C. Invasive measurement of arterial blood pressure through insertion of a 20-gauge cannula into the radial artery was performed after performing the Allen test in non-dominant hand. Baseline heart rate (HR) and mean arterial blood pressure (MABP) were recorded. Baseline PPI parameters were recorded using a Masimo pulse oximeter probe (Masimo Radical seven; Corp of Masimo, USA) placed in the patient’s index finger of the hand contralateral to the intravemous fluid infusion.

Before induction of anesthesia, patients were randomly allocated into two groups (magnesium sulfate and labetalol groups), using a computer- generated randomization table with 25 patients in each group.

The magnesium sulfate group (*n* = 25): received IV bolus dose of 40 mg/kg magnesium sulfate in 100 mL normal saline over 15 min then a minutes continuous infusion of 10–15 mg/kg/h was titrated untill the target mean arterial blood pressure (55–65 mmHg) was achieved and discontinued 10 min before the end of the surgery. Patients in the labetalol group (*n* = 25): received an IV bolus dose of 0.25 mg/kg labetalol in 100 mL normal saline over 15 min then continuous infusion of 0.5–1 mg/kg/h was titrated untill a target mean arterial blood pressure (55–65 mmHg) was achieved and discontinued 10 min before the end of the surgery.

### General anesthesia

Pre-oxygenation 3–5 min with 100% oxygen was administered then anesthesia was induced using 2 mg/kg IV propofol and 1.5 ug/kg IV fentanyl. Endotracheal intubation was facilitated with 0.15 mg/kg IV cisatracurium. Anesthesia was maintained with 1.5% isoflurane in 100% O_2_ and incremental doses of cisatracurium 0.03 mg/kg/h were given. Ventilation was adjusted to maintain the end tidal CO_2_ (ETCO_2_) at 30– 35 mmHg.

All patients were in the supine position with the head up 45°, and infiltration of the nasal mucosa by epinephrine at a dilution of 1:100,000 plus 2 mL of 1% lidocaine was performed by the surgeon. Normothermia was maintained using warm blankets and warm IV fluids. Intraoperative MABP of < 50 mmHg and bradycardia of < 50 beats/min were treated with ephedrine 5 mg and atropine (0.5 mg).

At the end of the surgery, the inhalational anesthetic was turned off and the muscle relaxant was reversed by neostigmine 0.05 mg/kg plus atropine 0.01 mg/kg. The patient was then extubated and transferred to the recovery room.

### Outcome variables

#### Intraoperative outcomes

- ▪ PPI by pulse oximeter was measured at basaline before induction, intubation, 5 and, 10 min after intubation and then every 10 min until the end of the surgery. The normal range is (0.02% –20%) [14].
- ▪ Mean arterial blood pressure and HR were recorded at basaline before induction, intubation, 5 and, 10 min after intubation and then every 10 min until the end of the surgery.
- ▪ The mean time to achieve the target mean arterial blood pressure (the time from the end of the bolus dose until the reach mean arterial blood pressure was 55–65 mmHg) in both groups was recorded.

#### Postoperative outcomes

1. Postoperative pain was evaluated using VAS score. It was assessed 30 min, 1 h, 2 h and 4 h postoperatively. IV paracetamol 1 gm was given every 6 h as a protocol for pain management and was started at the end of surgery. IV pethidine 1 mg/kg (rescue analgesic) was administered if VAS ≥ 4.
2. The time from the cessation of the infusion solution to the first call for pethidine (rescue analgesic) was recorded.
3. Total pethidine consumption.
4. Serum lactate levels were recorded at basaline and at 1 h after extubation. The normal serum lactate ranges from 4.5 to 19.8 mg/dL [15].

### Sample size

We compared the magnesium sulfate and labetalol- treated groups with respect to the PPI. The study consisted of two treated groups, effect size (f) = 1 and, power = 0.9 with a significance *P-*value at cut-off = 0.05, calculated using student’s t-test (unpaired and two-tailed) [16]. This analysis was done in R coding language, package “pwr” and function “pwr.t.test” [17]. Therfore, 25 patients per group were included in this study.

### Statistical analysis

Data were coded and entered using Statistical Package for Social Sciences (SPSS) version 26 (IBM Corp., Armonk, NY, USA). Data were summarized using mean and standard deviation for quantitative variables and frequencies (number of cases) and relative frequencies (percentages) for categorical variables. Comparisons between groups were performed using the unpaired t- test. To compare categorical data, the Chi square (χ2) test was performed. The exact test was used when the expected frequency was < 5. Statistical significant was set at *P* < 0.05. The confidence interval was determined at 95% intervals to represent the range of difference, bounded above and below the statistical means between the two groups.

## Results

**Fig. 1:**
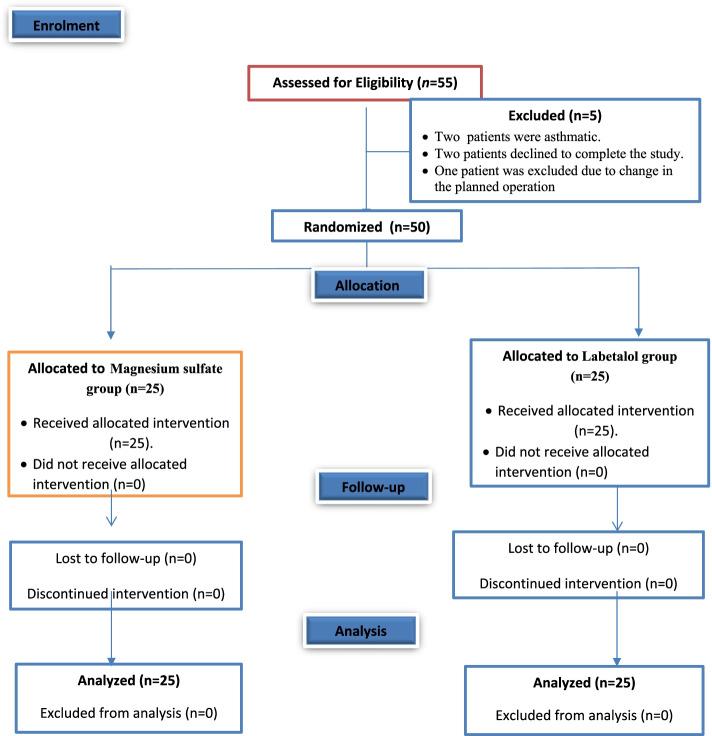
Consort flow chart

A total of fifty-five patients were prepared for the study; five patients were excluded from the study (two patients were asthmatic, two patients refused to complete the study, and one patient had a change in the planned operation) as presented in the CONSORT Statement for Reporting Trials (Fig. 1). Thus, a total of 50 patients were randomly allocated to two groups (25 patients each). Patient characteristics (age, sex, BMI, ASA I, and II) and operative data were comparable between the two groups (Table 1).

**Table 1:** Patients’ characteristics and operative data

	**Studied groups**		**t/x**^**2**^	***p*-value**
	**Magnesium sulfate group** **(*n* = 25)**	**Labetalol group** **(*n* = 25)**		
**Age(years)**	31.8 ± 5.6	32.9 ± 5.7	t = 0.67	0.50
**Sex**				
male	14 (56%)	13 (52%)	x^2^ = 0.08	0.77
female	11 (44%)	12 (48%)		
**ASA**				
**I**	13 (52%)	14 (56%)	x^2^ = 0.08	0.77
**II**	12 (48%)	11 (44%)		
**BMI (kg/m**^**2**^**)**	25.5 ± 2.6	25.2 ± 2.7	t = 0 .35	0.72
**Duration of surgery ( min)**	104.2 ± 7.1	104.1 ± 5.5	t = 0.09	0.93
**Type of surgery**				
• Endoscopic septoplasty	4 (16%)	5 (20%)	x^2^ = 0.36	1
• FESS	10(40%)	1144%)		
• Rhinoplasty	6 (24%)	a.(20%)		
• Septoturbanoplasty	5 (20%)	4 (16%)		

Data were expressed as mean ± SD, or No (%). *P* < 0.05 was significant. t = unpaired t- test. x^2^ = chi square test. *ASA* American Society of Anesthesiologist, *BMI* Body Mass Index and *FESS* Functional Endoscopic Sinus Surgery

The magnesium sulfate and labetalol groups were comparable in terms of the PPI values at baseline, intubation, and 5 min after intubation (*p* > 0.05). In addition, magnesium sulfate group showed significantly increased PPI values compared to the labetalol group (*p* < 0.001) (Table 2).

**Table 2:** PPI between the studied groups

PPI	Studied groups		Mean difference 95% (CI)	*P* value
	**Magnesium sulfate group** **(*n* = 25)**	**Labetalol group** **(*n* = 25)**		
**At Basal**	5.3 ± 0.3	5.1 ± 0.3	0.1 (-0.04 to 0.3)	0.12
**At intubation**	5.1 ± 0.5	4.9 ± 0.3	0.2 ( -0.05 to 0.4)	0.106
**At 5 min**	5 ± 0.3	4.8 ± 0.3	0.2 (-0.02 to0.4)	0.072
**At 10 min**	5 ± 0.4	4.4 ± 0.3	0.5 (0.3 -0.8)	< 0.001
**At 20 min**	4.9 ± 0.3	4 ± 0.4	0.9 (0.7 – 1.1)	< 0.001
**At 30 min**	5 ± 0.4	3.8 ± 0.3	1.2 (0.9- 1.4)	< 0.001
**At 40 min**	4.8 ± 0.3	3.6 ± 0.3	1.3 (1.1- 1.5)	< 0.001
**At 50 min**	4.6 ± 0.4	3.3 ± 0.3	1.3 (1.1- 1.5)	< 0.001
**At 60 min**	4.4 ± 0.3	3.1 ± 0.4	1.3 (1.1 – 1.5)	< 0.001
**At 70 min**	4.2 ± 0.3	2.8 ± 0.4	1.4 (1.1- 1.6)	< 0.001
**At 80 min**	4.1 ± 0.3	2.6 ± 0.4	1.5 (1.2 -1.7)	< 0.001
**At 90 min**	4 ± 0.4	2.5 ± 0.5	1.6 (1.3–1.8)	< 0.001
**At 100 min**	3.9 ± 0.3	2.4 ± 0.5	1.5 (1.2–1.7)	< 0.001
**At 110 min**	3.8 ± 0.3	2.5 ± 0.4	1.3 (1.1–1.5)	< 0.001

*PPI* Peripheral Prefusion Index Data were expressed as mean ± SD, *P* < 0.05was significant. *CI* Confidence Interval

**Fig. 2:**
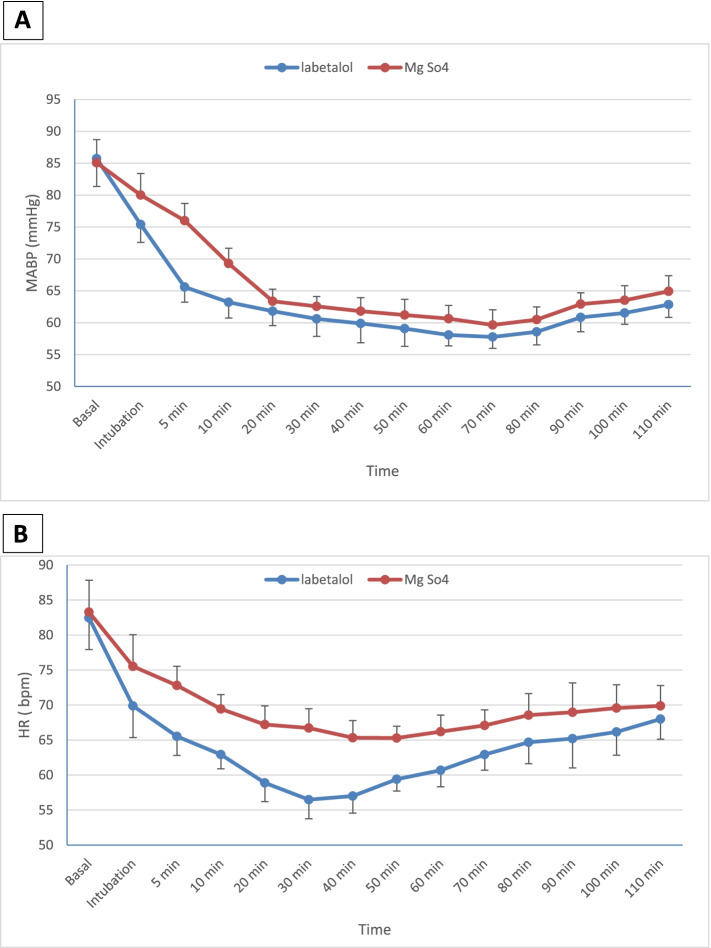
**A** = Mean arterial blood pressure (MABP) (mmHg) between studied groups at the measured time points.** B** = Mean heart rate (HR) beat/minute (bpm) between studied groups at the measured time points. Mean ± SD

Regarding the mean arterial blood pressure, both groups were comparable at baseline (*p* > 0.05). In contrast, magnesium sulfate group showed a statistically significant increase in MABP values compared with the labetalol group (*P* < 0.05) (Fig. 2A). The time to reach the target MABP was significantly prolonged in the magnesium sulfate group compared to the labetalol group ([21.6 ± 1.7 vs 6.9 ± 1.5] min, *p* < 0.001, mean difference = 14.7[95% CI, 13.7–15.6]) (Table 3).

**Table 3:** Time to reach the target mean arterial blood pressure, serum lactate level and analgesic parameters between groups

		**Studied groups**		**Mean difference (95% CI)**	***P* value**
		**Magnesium sulfate group (*n* = 25)**	**Labetalol group (*n* = 25)**		
**Time to reach the target mean arterial blood pressure (min.)**		21.6 ± 1.7	6.9 ± 1.5	14.7(13.7 **–** 15.6)	< 0.001
**Serum lactate level (mg/dL)**	**Basal**	10.6 ± 3.2	11.8 ± 3.9	-1.1 (-3.2 to 0.8)	0.24
	**After 1 h**	11.1 ± 3	12.6 ± 3.6	-1.5 (-3.4to 0.3)	0.1
**Time to the first call of pethidine (min.)**		113.1 ± 5.2	28.2 ± 1.5	84.8 (82.6 -87.1)	< 0.001
**Total Pethidine Consumption (mg)**		64.2 ± 4.7	71.04 ± 6.2	-6.7 (-9.9 to -3.6)	< 0.001

The data were expressed as mean ± SD. *P* < 0.05was significant. *CI* Confidence Interval

Heart rate values were statistically highly significant lower in the labetalol group than magnesium sulfate group starting from intubation until 110 min after anesthesia induction (*p* < 0.001), and both groups were comparable at baseline values (*p* > 0.05) (Fig. 2B).

Serum lactate levels were comparable between the two groups at baseline, and 1 h postoperatively (*p* > 0.05) (Table 3).

**Fig. 3:**
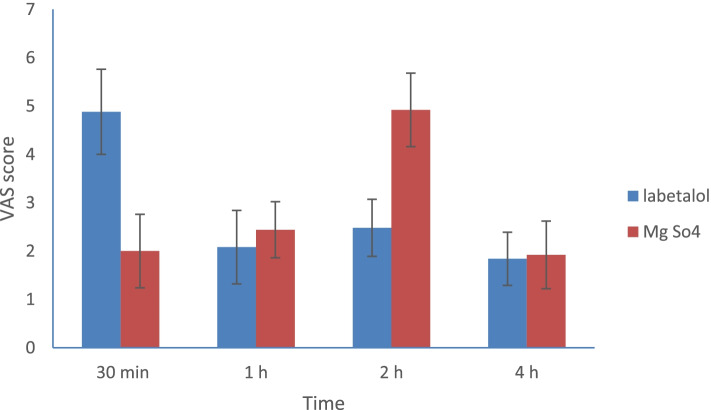
Mean visaual analogue scale (VAS) scores between the studied groups. Mean ± SD

Regarding postoperative pain, the magnesium sulfate group showed significantly lower VASscores up to 2 h compared to the labetalol group (*p* < 0.001) (Fig. 3). The time to first call of pethidine was significantly prolonged in the magnesium sulfate group compared to the labetalol group ([113.1 ± 5.2 vs 28.2 ± 1.5] min, *p* < 0.001, mean difference = 84.8[95% CI, 82.6 –87.1]) and the total pethidine consumption was significantly lower in magnesium sulphate group compared to labetalol group ([64.2 ± 4.7 vs 71 ± 6.2] mg, *P* < 0.001 mean difference = -6.7 [ 95% CI, -9.9 to -3.6]) (Table 3).

## Discussion

The current study demonstrated that magnesium sulfate infusion maintains wider PPI values compared to labetalol and was significantly associated with higher intraoperative mean arterial blood pressure and HR than labetalol in healthy patients. It is important to maintain peripheral perfusion even in healthy patients, moreover in particularly ill patients.

To the best of our knowledge, this is the first study to compare the effects of magnesium sulfate and labetalol on PPI and postoperative pain during induced hypotension in nasal surgery.

PPI depends mainly on vascular tone as peripheral vasodilatation increases PPI, while vasoconstriction decreases PPI [18]. Zayed et al. [16] concluded that nitroglycerin was associated with a higher PPI than labetalol during induced hypotension in patients undergoing sinus endoscopic surgery and they explained that vasodilator drugs such as nitroglycerin enhance blood flow to the microcirculation, while IV infusion of labetalol antagonizes the non-selective B adrenergic receptors by seven times more than the postsynaptic α1 adrenergic receptors. B1 adrenergic receptor antagonists lower HR, while α1 adrenergic receptor antagonists decrease vascular resistance, which leading to vasodilation, and which is clinically correlated with the present study results considering magnesium sulfate as a vasodilator drug. Magnesium sulfate stimulates the synthesis of prostacyclin I_2_ (PGI_2_) in vascular cells, subsequently regulating vascular tone directly. Magnesium sulfate induces vascular relaxation because of its Ca +  + antagonistic action [19].

In the present study, the time to reach the target MABP in the magnesium group was highly statistically significant longer (21.6 ± 1.7 min) than labetalol group (6.9 ± 1.5 min).

Chhabra et al. [20] reported that the mean time to reach MABP in magnesium group was 21.32 ± 4.65 min when comparing the effect of dexmedetomidine and magnesium sulfate on inducing hypotension in endoscopic sinus surgery**.** However, in the study by Patel et al. [21], the time to achieve a target arterial blood pressure of 140/90 mmHg in labetalol was 12.63 ± 7.19 min which might be related to our results; however, labetalol might take a longer time than in our patients as their study was conducted in hypertensive pregnant mothers.

The results of this study revealed that magnesium sulfate was superior to labetalol in maintaining the PPI values. Therefore, PPI could serve to detect better- induced hypotension.

Højlund et al. [22] found that PPI is a reliable parameter for tracing acute changes in hemodynamics during general anesthesia and there was a strong correlation between changes in PPI and (MABP, cardiac output, and stroke volume), and which can predict hypotension and decrease cardiac output during general anesthesia. In the same context, Choudhary et al. [23] reported that PPI can be used as an additional parameter to evaluate the hemodynamic response to laryngoscopy during intubation as it had a good correlation with mean arterial blood pressure and HR.

Serum lactate is a reliable biochemical marker for monitoring tissue perfusion clinically, but is a late marker reflecting the process of anaerobic metabolism [24]. Therefore, in this study, there was no difference between basal readings and 60 min after extubation between both groups as the induced hypotension state is different from shock.

The analgesic effect of labetalol has been discussed in the previously literature [25, 26]. However, to date, the present study is the first randomized clinical study to determine the effect of labetalol compared to magnesium sulfate in pain management after nasal surgery. This study revealed that magnesium sulfate was superior to labetalol in reducing pain intensity using VAS score up to 2 h postoperatively, the time to first call of pethidine in magnesium sulfate group was (113.1 ± 5.2 min) compared to labetalol group (28.2 ± 1.5 min) and the total pethidine consumption was significantly reduced in the magnesium sulfate group (64.2 ± 4.7 mg) compared to the labetalol group (71 ± 6.2 mg). Elsersy et al. [7] reported in their study results that intraoperative magnesium sulfate infusion reduced the pain scores in patients undergoing functional endoscopic surgery. In addition, Chen et al. [27] revealed that magnesium sulfate was useful in decreasing pain intensity in the early stages after laparoscopic cholecystectomy.

The present study was conducted on healthy adult volunteers not suffering from any peripheral vascular diseases to avoid bias of the results and to take it as the standard for the best hypotensive agent with wider PPI. Therefore, we recommend further studies in the elderly and in patients with peripheral vascular diseases.

### Limitations

The first limitation was the lack of previous studies in this field of the present study. Second, we did not assess the surgical field because our target mean arterial blood pressure was 55–65 mmHg, which has been approved in many previous clinical trials to produce a clear surgical field [16, 28, 29]. Therefore, further studies are warranted.

## Conclusions

Magnesium sulfate maintains wider PPI and offers better postoperative pain relief compared to labetalol during induced hypotension in nasal surgeries. PPI can be used to detect better -induced hypotensive agent during general anesthesia.

## Supplementary Information

**Additional file 1.**

## Data Availability

The data sets during the current study are available from the corresponding author on reasonable request.

## Abbreviations

PPI: Peripheral perfusion index
MABP: Mean arterial blood pressure
HR: Heart rate
CI: Confidence interval
ASA: American Society of Anesthesiologists
BMI: Body mass index
VAS: Visual analogue scale
IV: Intravenous

## References

[1] Erdem AF, Kayabasoglu G, Tas Tuna A, Palabiyik O, Tomak Y, Beyaz SG (2016). Effect of controlled hypotension on regional cerebral oxygen saturation during rhinoplasty: a prospective study. J Clin Monit Comput.

[2] Slack WK, Walther WW (1963). Cerebral circulation studies during hypotensive anaesthesia using radioactive xenon. Lancet.

[3] Falotico JM, Shinozaki K, Saeki K, Becker LB (2020). Advances in the approaches using peripheral perfusion for monitoring hemodynamic status. Front Med (Lausanne).

[4] Hasanin A, Mukhtar A, Nassar H (2017). Perfusion indices revisited. J Intensive Care.

[5] Lima AP, Beelen P, Bakker J (2002). Use of a peripheral perfusion index derived from the pulse oximetry signal as a noninvasive indicator of perfusion. Crit Care Med.

[6] Kumar A, Nadkarni AV (2017). The variability of perfusion index as a new parameter in different types of anaesthesia techniques and its correlation with surgical stress and recovery from anesthesia: an observational clinical Study. JMSCR..

[7] Elsersy HE, Metyas MC, Elfeky HA, Hassan AA (2017). Intraoperative magnesium sulphate decreases agitation and pain in patients undergoing functional endoscopic surgery: A randomised double-blind study. Eur J Anaesthesiol.

[8] Ryu JH, Sohn IS, Do SH (2009). Controlled hypotension middle ear surgery. A comparison between remifentanil and magnesium sulfate. Br J Anaesth..

[9] Banerjee S, Jones S. Magnesium as an Alternative or Adjunct to Opioids for Migraine and Chronic Pain: A Review of the Clinical Effectiveness and Guidelines [Internet]. Ottawa (ON): Canadian Agency for Drugs and Technologies in Health; 2017. 29334449

[10] Shin HJ, Na HS, Do SH (2020). Magnesium and pain. Nutrient.

[11] Elshmaa NS, Ezz HAA, Younes A (2017). The efficacy of labetalol versus nitroglycerin for induction of controlled hypotension during sinus endoscopic surgery. A prospective, double blind and randomized study. J Clin Anesth..

[12] Martin LJ, Piltonen MH, Gauthier J, Convertino M, Acland EL, Dokholyan NV (2015). Differences in the Antinociceptive effects and binding properties of propranolol and Bupranolol enantiomers. J Pain.

[13] McCormack HM, Horne DJ, Sheather S (1988). Clinical applications of visual analogue scales: a critical review. Psychol Med.

[14] Uygur O, Koroglu OA, Levent E, Tosyali M, Akisu M, Yalaz M (2019). The value of peripheral perfusion index measurments for early detection of critical cardiac defects. Pediatr Neonatol.

[15] Odom  SR, Talmor  D (2016). What is the meaning of a high lactate? What are the implications of lactic acidosis?. Deutschman CS, Neligan PJ, eds. Evidence-Based Practice of Critical Care.

[16] Zayed M, Nassar H, Hasanin A, Saleh AH, Hassan P, Saad D (2020). Effects of nitroglycerin versus labetalol on peripheral perfusion during deliberate hypotension for sinus endoscopic surgery: a randomized, controlled, double-blinded trial. BMC Anesthesiol.

[17] Cohen J. Statistical Power Analysis for the Behavioral Sciences (2nd ed.). Routledge: Lawrence Erlbaum Associates; 1988. 10.4324/9780203771587.

[18] Rasmy I, Mohamed H, Nabil N, Abdalah S, Hasanin A, Eladawy A (2015). Evaluation of perfusion index as a predictor of vasopressor requirement in patients with severe sepsis. Shock.

[19] Satake K, Lee JD, Shimizu H, Uzui H, Mitsuke Y, Yue H (2004). Effects of magnesium on prostacyclin synthesis and intracellular free calcium concentration in vascular cells. Magnes Res.

[20] Chhabra A, Saini P, Sharma K, Chaudhary N, Singh A, Gupta S (2020). Controlled hypotension for FESS: A randomised double-blinded comparison of magnesium sulphate and dexmedetomidine. Indian J Anaesth.

[21] Patel P, Koli D, Maitra N, Sheth T, Vaishnav P (2018). Comparison of efficacy and safety of intravenous labetalol versus hydralazine for management of severe hypertension in pregnancy. J Obstet Gynaecol India.

[22] Højlund J, Agerskov M, Clemmesen CG, Hvolris LE, Foss NB (2020). The Peripheral Perfusion Index tracks systemic haemodynamics during general anaesthesia. J Clin Monit Comput.

[23] Choudhary VK, Rastogi B, Singh VP, Ghalot S, Dabass V, Ashraf S (2018). Comparison of hemodynamic responses along with perfusion index to tracheal intubation with Macintosh and McCoy laryngoscopes. Int J Res Med Sci.

[24] Janotka M, Ostadal P (2021). Biochemical markers for clinical monitoring of tissue perfusion. Mol Cell Biochem.

[25] Margaria E, Gagliardi M, Palieri L, Treves S, Fanzago E (1983). Analgesic effect of peridural labetalol in the treatment of cancer pain. Int Clin Pharmacol Therap Toxicol.

[26] Xiao C, Zhou C, Atlas G, Delphin E, Ye J (2008). Labetalol facilitates GABAergic transmission in rat periaqueductal gray neurons via antagonising B1-adrenergic receptors- possible mechanism underlying labetalol induced analgesia. Brain Res.

[27] Chen C, Tao R (2018). The impact of magnesium sulfate on pain control after laparoscopic cholecystectomy: a meta-analysis of randomized controlled studies. Surg Laparosc Endosc Percutan Tech.

[28] Bayoumy AA, Abo Zeid GS (2020). El Deek AM and Elbeialy MA : Comparative study between magnesium sulphate and dexmedetomidine in controlled hypotension during functional endoscopic sinus surgery: a prospective randomized study. Ain-Shams J Anesthesiol.

[29] Mahajsan L, Singh AP, Chawla S, Gill S (2020). Premedication for induced hypotension in functional endoscopic sinus surgeries: Intravenous dexmedetomidine infusion vs oral metoprolol vs placebo: A comparative study. Anesth Essays Res.

